# The Effect of pH on the Viscoelastic Response of Alginate–Montmorillonite Nanocomposite Hydrogels

**DOI:** 10.3390/molecules29010244

**Published:** 2024-01-02

**Authors:** Haniyeh Malektaj, Aleksey D. Drozdov, Elham Fini, Jesper de Claville Christiansen

**Affiliations:** 1 Department of Materials and Production, Aalborg University, Fibigerstraede 16, 9220 Aalborg, Denmark; aleksey@mp.aau.dk (A.D.D.); jc@mp.aau.dk (J.d.C.C.); 2 School of Sustainable Engineering and Built Environment, Arizona State University, 660 S College Ave, Tempe, AZ 85281, USA; efini@asu.edu

**Keywords:** alginate, hydrogel, montmorillonite, nanocomposite, mechanical properties

## Abstract

Ionically cross-linked alginate hydrogels are used in a wide range of applications, such as drug delivery, tissue engineering, and food packaging. A shortcoming of these gels is that they lose their strength and degrade at low pH values. To develop gels able to preserve their integrity in a wide range of pH values, Ca-alginate–montmorillonite nanocomposite gels are prepared, and their chemical structure, morphology, and mechanical response are analyzed. As the uniformity of nanocomposite gels is strongly affected by concentrations of MMT and CaCl_2,_ it is revealed that homogeneous gels can be prepared with 4 wt.% MMT and 0.5 M CaCl_2_ at the highest. The viscoelastic behavior of nanocomposite gels in aqueous solutions with pH = 7 and pH = 2 is investigated by means of small-amplitude compressive oscillatory tests. It is shown that Ca-alginate–MMT nanocomposite gels preserve their integrity while being swollen at pH = 2. The experimental data are fitted by a model with only two material parameters, which shows that the elastic moduli increase linearly with a concentration of MMT at all pH values under investigation due to formation of physical bonds between alginate chains and MMT platelets. The presence of these bonds is confirmed by ATR-FTIR spectroscopy. The morphology of nanocomposite gels is studied by means of wide-angle X-ray diffraction, which reveals that intercalation of polymer chains between clay platelets increases the interlayer gallery spacing.

## 1. Introduction

Applications of biopolymeric hydrogels in controlled drug delivery have recently received significant attention [1,2,3,4]. The pH sensitivity of gels is crucial for the design of drug release systems to treat gastric diseases, prevent infections, or deliver cancer therapy in the acidic environment of tumors [5,6,7,8]. The shortcomings of some biopolymeric hydrogels are their decreased stability, degradation, and erosion in aqueous solutions with low pH values. These phenomena affect their ability to retain drugs and reduce their effectiveness as drug release systems.

Ca-alginate hydrogels are commonly used materials for drug delivery with versatile applications [9,10,11]. However, the range of their applications is limited to weak acidic conditions with pH > 5.5 only [12,13]. When Ca-alginate gels are exposed to strong acidic environments, calcium ions that cross-link alginate chains in the gel matrix are replaced with protons, which leads to disintegration of the gel structure [14]. This hinders applications of Ca-alginate gels in pH below the pK_a_ of alginate chains (the value of pK_a_ belongs to the interval between 3.6 for mannuronic acid and 3.8 for guluronic acid [15]).

Montmorillonite (MMT) is a natural clay mineral known for its high surface area and its capacity for cation exchange. These properties make it valuable to modulate the release of pharmaceuticals [16,17,18]. The incorporation of MMT into biopolymer gels improves the moisture barrier properties [19], enhances the pervaporation dehydration performance [20,21] and the entrapment efficiency of drugs [22], and enhances the mechanical properties of alginate gels [19,23,24]. The biocompatibility of MMT and alginate makes their nanocomposites suitable for biomedical applications [11,25]. Chemical modification of MMT clay before mixing with biopolymers enhances their biocompatibility, the mechanical strength of the composites, and their structural integrity [20,21].

The objective of this study is twofold. Our first purpose is to find suitable ranges of MMT and CaCl_2_ concentrations that allow homogeneous alginate–MMT nanocomposite gels to be prepared with repeatable mechanical responses. The uniformity of the gels is important because enhancement of the mechanical properties of MMT biopolymer gels depends strongly on the uniform dispersion of clay [20]. When an alginate gel contains relatively high amounts of MMT and CaCl_2_ (which both serve as cross-linkers in its polymer networks), the gel becomes inhomogeneous [26]. Nanocomposite biopolymer gels were prepared in previous studies [27,28,29] with concentrations of MMT up to 5 wt.%.

Secondly, cross-linking of alginate with MMT and CaCl_2_ leads to the formation of two different types of physical bonds: between alginate chains and MMT platelets, and between alginate chains bridged by Ca^2+^ ions [30]. Our objective is to investigate the difference between these bonds and their influence on the mechanical response characterized by dynamic mechanical analysis (DMA). For this purpose, alginate–MMT hydrogels were prepared with different MMT concentrations at pH = 7. Afterwards, the gels were immersed in aqueous solutions with pH = 2. It is known that bonds between alginate chains and Ca^2+^ ions are partially broken upon immersion of the gels in acidic environments with pH = 2 [14].

To analyze the effect of pH of aqueous solutions on the viscoelastic response of nanocomposite hydrogels, we focus on solutions with pH = 2 and pH = 7 (below and above the pK_a_ of alginate chains). pH = 2 was chosen because this pH value is typical for biomedical applications related to gastric diseases. Experimental data for the storage and loss moduli in small-amplitude oscillatory tests (the frequency-sweep mode) are matched simultaneously by a simple model in linear viscoelasticity with two material parameters. Fitting shows that the elastic modulus of the polymer network increases linearly with concentration of MMT platelets at both pH values under investigation, whereas a measure of inhomogeneity of the network remains practically independent of the concentration of nanofiller. An increase in the elastic modulus of the network with mass fraction of MMT at normal pH = 7 is explained by electrostatic interactions between negatively charged COO^−^ groups of alginate chains and positively charged edges of clay platelets. The growth in the elastic modulus with concentration of MMT at pH = 2, when all the carboxyl groups are protonated and electrostatic interactions disappear, is ascribed to formation of hydrogen bonds between neutral COOH groups of alginate chains and OH groups attached to the surfaces of MMT platelets.

The novelty of this study consists of the following: (i) limits are determined on concentrations of MMT clay and Ca^2+^ ions, within which nanocomposite gels remain homogeneous on the one hand and preserve their integrity at low pH values on the other; (ii) it is demonstrated that the elastic modulus of nanocomposite gels increases with mass fraction of MMT clay in aqueous solutions with all pH values under consideration; (iii) to explain this growth, a model is proposed that presumes replacement of electrostatic interactions between negatively changed carboxyl groups of alginate chains and positively charged edges of platelets at high pH values with hydrogen bonds between neutral carboxyl groups and OH groups attached to the surfaces of MMT platelets at low pH values.

## 2. Results

### 2.1. Preparation of Uniform Nanocomposite Gels

**Figure 1:**
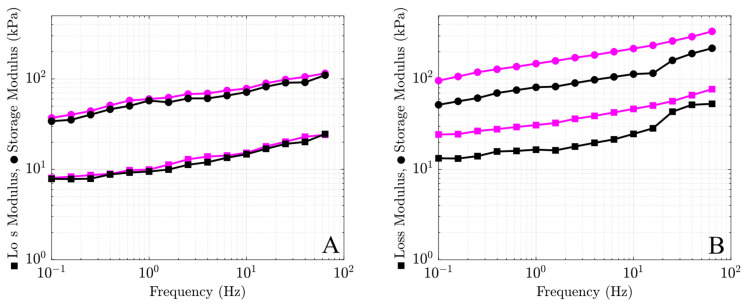
Storage modulus *E*′ (circles) and loss modulus *E*″ (squares) versus frequency *f* for alginate–MMT hydrogels cross-linked with (**A**) 4 wt.% of MMT and 0.5 M CaCl_2_ and (**B**) 4.5 wt.% of MMT and 1.5 M CaCl_2_ in aqueous solutions with pH = 7. Each figure shows observations on two samples (black and purple lines) with identical preparation conditions.

To choose proper concentrations of MMT and CaCl_2_ that ensure uniformity of nanocomposite gels, several series of gels were prepared with concentrations of MMT clay ranging from 1 to 4.5 wt.% and concentrations of CaCl_2_ ranging from 0.5 to 1.5 M. Figure 1A shows good repeatability of data in the DMA test on two samples prepared with 4 wt.% MMT and 0.5 M CaCl_2_. When the content of MMT and CaCl_2_ went beyond 4 wt.% and 0.5 M, respectively, poor repeatability of observations in the DMA test was observed in Figure 1B, which shows the storage modulus *E*′ and the loss modulus *E*″ of two samples prepared with 4.5 wt.% MMT and 1.5 M CaCl_2_. Relatively large deviations between the data in Figure 1B can be attributed to the fact that the homogeneity of samples decreases with an increase in MMT and CaCl_2_ concentration [31,32]. The inhomogeneity is caused by the disk-like morphology of MMT clay platelets, which serve as barriers that block the gel and prevent diffusion of the CaCl_2_ [26]. Moreover, at concentrations of MMT above 4 wt.%, clay platelets and their stacks interact with each other, which results in a substantial reduction in the stiffness and strength of nanocomposites [33].

In what follows, the nanocomposite gels prepared with concentrations of 1, 3, and 4 wt.% MMT and 0.5 M CaCl_2_ are analyzed.

### 2.2. ATR-FTIR Spectroscopy

**Figure 2:**
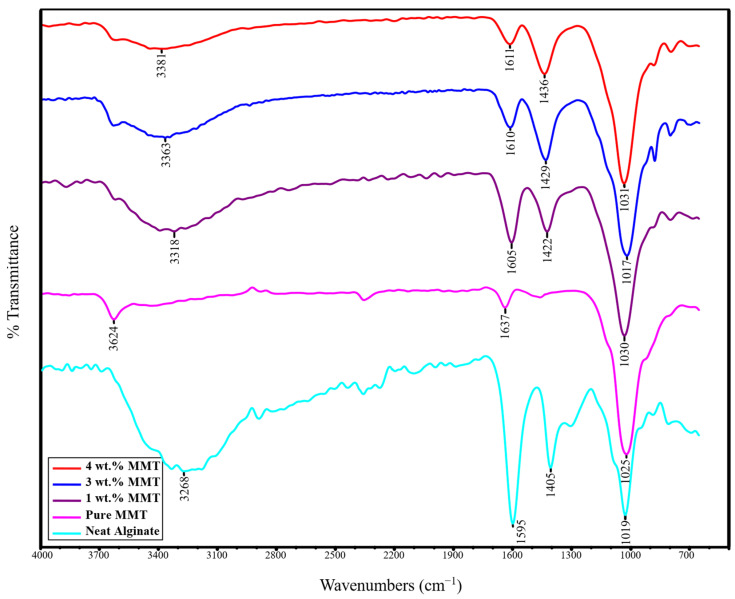
FTIR spectra of alginate, MMT, and Ca-alginate/MMT hydrogels with 1, 3, and 4 wt.% MMT.

To evaluate interactions between the MMT platelets and the alginate chains, attenuated total reflection-Fourier-transform infrared spectroscopy (FTIR) analysis was performed. The FTIR spectra of alginate, MMT, and alginate–MMT nanocomposite hydrogels with 1, 3, and 4 wt.% MMT are presented in Figure 2.

The absorption bands of MMT are assigned as follows: 3624 cm^−1^ corresponds to OH stretching vibration in Si–OH and Al–OH bonds. The band at 1637 cm^−1^ corresponds to the H–O–H bending vibration of adsorbed water, and the peak at 1025 cm^−1^ corresponds to Si–O stretching [34,35].

Sodium alginate is characterized by asymmetric and symmetric stretching vibrations at 1595 cm^−1^ and 1405 cm^−1^ due to carboxylic acid and at 1019 cm^−1^ for oxygen stretching in a cyclic ether bridge. The band at 3268 cm^−1^ corresponds to OH stretching vibration [34].

The FTIR spectrum of the nanocomposite hydrogels revealed that the chemical structures of the nanocomposite hydrogels are similar to alginate, which is the major fraction in the nanocomposite [34]. Comparing the alginate–MMT gels and the pristine MMT, it can be concluded that the Si–OH stretching of MMT at 3624 cm^−1^ disappears in the spectra of the nanocomposite gels, confirming that active sites of the polymer matrix interact with MMT. This phenomenon is explained in Refs. [20,21] by hydrogen bonding between the silanol hydroxyl groups and alginate carboxyl and hydroxyl groups.

The other result showing interaction of MMT with alginate chains is that the COOH alginate stretching bands at 1595 cm^−1^ and 1405 cm^−1^ shifted to higher values (see Table 1) in the nanocomposite gels. This shift is caused by interactions between OH groups attached to MMT platelets and carboxyl groups bound to alginate chains [36].

### 2.3. X-ray Diffraction

**Figure 3:**
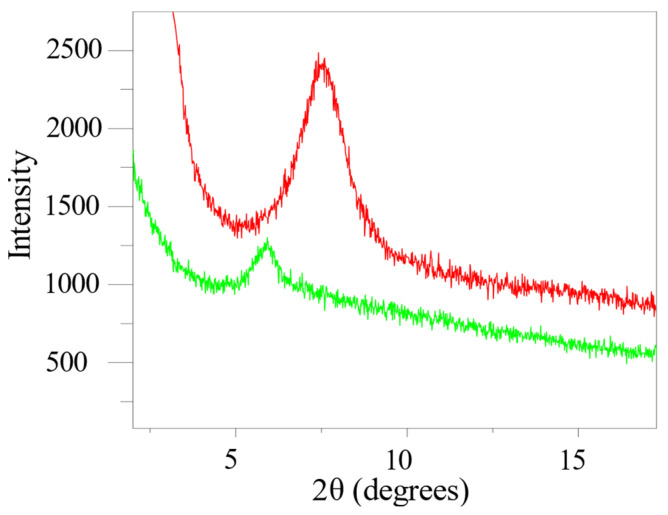
XRD patterns of pure MMT (red) and Ca-alginate/MMT nanocomposite hydrogel (green).

The degree of intercalation of MMT clay platelets in alginate–MMT nanocomposite hydrogel is investigated by means of wide-angle X-ray diffraction (XRD). The results are presented in Figure 3. It appears from the XRD pattern of pure MMT that the diffraction characteristic peak of the MMT plane [001] is at 2θ = 7.34° (d = 12.03Å), which coincides with the value reported in Ref. [29]. It is known that intercalation of polymer chains into the clay stacks increases the interlayer spacing of the clays, leading to a shift in the diffraction peak towards lower angle values. Figure 3 shows that the characteristic peaks of MMT 2θ changed from 7.34° to 5.91° (d = 14.92 Å) for nanocomposite hydrogels. The expanded gallery distance in modified MMT makes polymer molecules enter into the MMT gallery more easily [37]. The decrease in the intensity of the diffraction characteristic peak of the MMT plane [001] most likely indicates the disordered intercalated structure [37,38].

### 2.4. Mechanical Properties of Alginate–MMT Hydrogels

**Figure 4:**
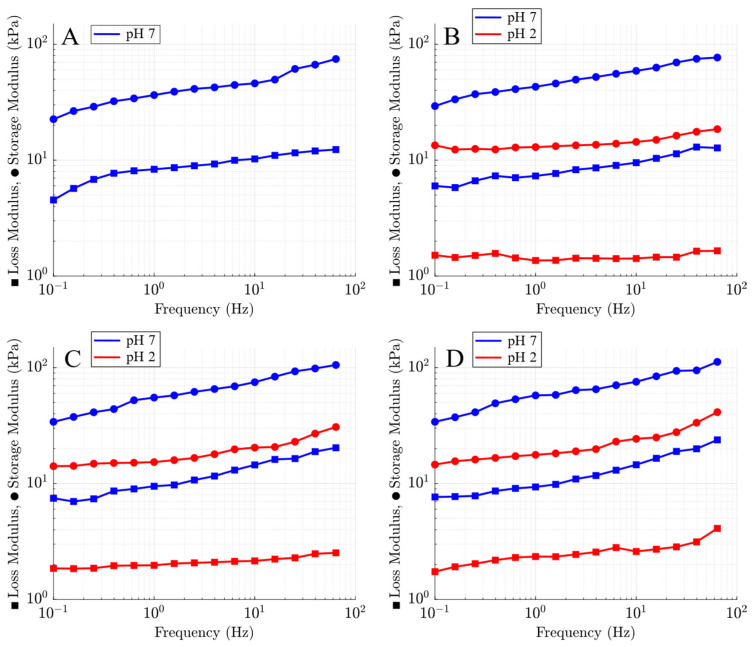
Storage modulus *E*′ (circles) and loss modulus *E*″ (squares) versus frequency *f* for alginate–MMT gels with (**A**) 0, (**B**) 1, (**C**) 3, and (**D**) 4 wt.% of MMT in aqueous solutions with pH = 7 and pH = 2.

The storage modulus (*E*′) and loss modulus (*E*″) of alginate–MMT hydrogels (with 0, 1, 3, and 4 wt.% MMT concentrations), immersed in solutions of pH = 7 or pH = 2, were determined by means of small-amplitude compressive oscillatory tests in the frequency-sweep mode. The influence of frequency *f* on these moduli is demonstrated in Figure 4.

The storage *E*′ and loss *E*″ moduli of the alginate–MMT nanocomposite hydrogels at pH =7 (Figure 4B–D) are higher than those of the neat alginate hydrogel (Figure 4A) in the entire frequency range due to formation of physical bonds between alginate chains and MMT platelets. The storage modulus *E*′ and loss modulus *E*″ of the nanocomposite gels increase with the concentration of MMT. The storage modulus *E*′ and loss modulus *E*″ of all samples in aqueous solutions with pH = 2 are lower than those in water with pH = 7.

**Figure 5:**
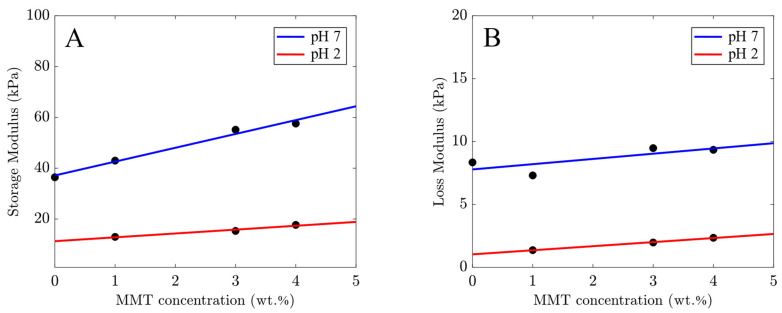
(**A**) Storage modulus *E*′ and (**B**) loss modulus *E*″ at frequency *f* = 1 Hz versus concentrations of MMT in aqueous solutions with pH = 7 and pH = 2. Circles: experimental data. Solid line: their approximation by a linear function.

Alginate chains in Ca-alginate–MMT nanocomposite gels are cross-linked by two types of bonds: between Ca^2+^ and alginate chains, and between MMT platelets and polymer chains. To understand which bonds are broken at pH = 2, the storage modulus *E*′ and the loss modulus *E*″ measured at frequency *f* = 1 Hz are plotted versus MMT concentration in aqueous solutions with pH = 7 and pH = 2. The results are depicted in Figure 5, where the experimental data are approximated by the linear functions. This figure shows that the slopes of both dependencies (for the storage modulus *E*′ and the loss modulus *E*″) are relatively close at pH = 2 and pH = 7.

**Figure 6:**
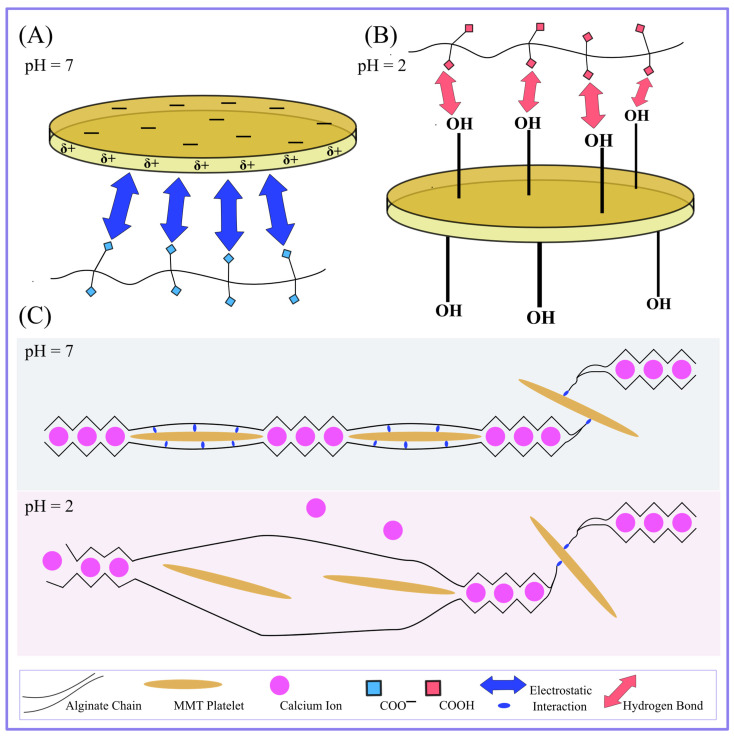
(**A**) Schematic presentation of the electrostatic links between the positively charged edge of MMT and the negatively charged alginate chains at pH = 7, and (**B**) hydrogen bonds between the OH groups of MMT and protonated COOH groups of alginate chains at pH = 2. (**C**) By lowering the pH from 7 to 2, the weak “egg-box” structure is broken; consequently, the electrostatic connection between MMT and alginate chains is broken.

The storage and loss moduli of alginate–MMT nanocomposite hydrogels characterize the structure of their polymer networks because they are proportional to concentrations of physical bonds between chains. These bonds are formed when alginate chains are cross-linked by Ca^2+^ ions and MMT platelets (negatively charged ionized carboxyl groups of alginate chains form electrostatic bonds with positively charged edges of clay platelets) under preparation of the gels at pH = 7 (see Figure 6). Reduction in pH from pH = 7 to pH = 2 causes notable changes in the structure of the polymer network. Under strongly acidic conditions, the concentration of bonds formed by Ca^2+^ ions decays due to release of these ions, whereas all the carboxyl groups of alginate chains become protonated. As a result, electrostatic interactions between chains and clay platelets are replaced by hydrogen bonds formed between neutral COOH groups of alginate chains and OH groups attached to the surfaces of MMT platelets (Figure 6).

To assess how this phase transition affects the mechanical properties of nanocomposite gels, the experimental data depicted in Figure 4 were fitted by means of the model in linear viscoelasticity proposed in Ref. [39]. The model treats a nanocomposite gel as a transient network of flexible polymer chains linked by permanent and temporary (reversible) bonds. Temporary bonds break and reform at random instants. Rearrangement of these bonds is driven by thermal fluctuations. Each temporary bond is characterized by its dimensionless (normalized by the thermal energy) activation energy for breakage *v*. The system of temporary bonds between chains is presumed to consist of bonds with activation energies *v* that vary from zero to infinity [40]. The ratio of the number of temporary bonds with activation energy *v* belonging to the interval [*v*, *v* + *dv*] to the total number of temporary bonds in the network reads *f*(*v*)*dv*, where *f*(*v*) stands for the probability density to find a reversible bond with activation energy *v*. The following quasi-Gaussian expression is adopted for the probability density:

(1) $$f\left(v\right)=f_{0}.\exp\left(-\frac{v^{2}}{2\;\Sigma^{2}}\right)$$

An advantage of Equation (1) is that it involves the only parameter Σ that is treated as a measure of inhomogeneity of a polymer network, while the coefficient *f*_0_ is determined from the normalization condition.

The rate of breakage *Γ*(*v*) for a temporary bond with activation energy *v* is determined by the Eyring equation

(2) $$\Gamma \left(v\right)=\Gamma_{0}.\exp\;\left(-v\right)$$

where the pre-factor *Γ*_0_ stands for the attempt rate for breakage of temporary bonds.

The storage modulus *E*′(*ω*) and the loss modulus *E*″(*ω*) are determined by the formulas

(3) $$E^{\prime }\left(\omega \right)=E\int_{0}^{\infty }f\left(v\right)\frac{\left(1-\kappa \right)\;\Gamma^{2}\left(v\right)+\omega^{2}}{\Gamma^{2}\left(v\right)+\omega^{2}}dv$$

(4) $$E^{\prime \prime }\left(\omega \right)=E\int_{0}^{\infty }f\left(v\right)\frac{\kappa \;\Gamma \left(v\right)\;\omega }{\Gamma^{2}\left(v\right)+\omega^{2}}dv\;\;$$

where *E* denotes the elastic modulus (which is proportional to the total number of permanent and temporary bonds between chains), and *κ* stands for the ratio of the number of temporary bonds to the total number of bonds between polymer chains. A detailed derivation of Equations (3) and (4) is provided in Ref. [39].

Given an angular frequency ω=2πf, Equations (1)–(4) involve four adjustable parameters: (i) *E* stands for the elastic modulus of a nanocomposite gel, (ii) κ is the ratio of the number of temporary bonds in the network to the total number of bonds, (iii) Σ characterizes the distribution of temporary bonds with various activation energies, and (iv) *Γ*_0_ is the attempt rate for breakage of temporary bonds. These coefficients are found by fitting the experimental data reported in Figure 4.

To simplify the analysis, we presume *Γ*_0_ to be independent of pH and mass fraction of MMT clay. *Γ*_0_ is set to 15.000 s^−1^ in numerical simulation. In the matching procedure, we use the values Σ = 6.9 and 13.5 for pH = 7 and pH = 2, respectively. These values are determined from the condition of the best fit of the experimental data on samples with 1 wt.% of MMT. The strong decay in Σ with pH confirms that the inhomogeneity of nanocomposite gels increases at pH = 2 due to the breakage of bonds between chains and replacement of electrostatic interactions between MMT platelets and alginate chains with hydrogen bonds. The remaining two parameters, *E* and *κ*, are determined by approximating each set of data in Figure 4 separately.

**Figure 7:**
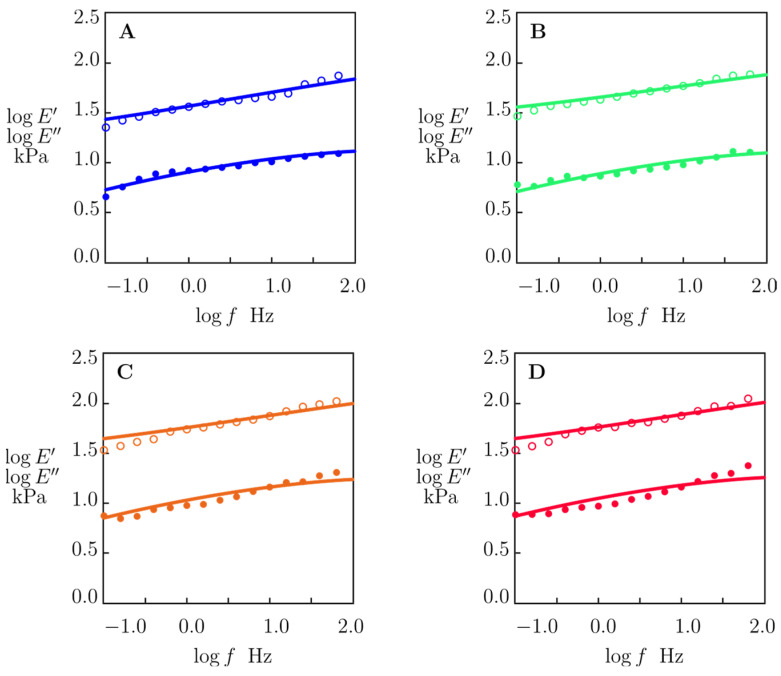
Storage modulus *E*′ (◦) and loss modulus *E*″ (•) versus frequency *f*. Symbols: experimental data in small-amplitude oscillatory tests at room temperature on alginate–MMT nanocomposite gels with various concentrations of MMT swollen in water with pH = 7. (**A**) 0, (**B**) 1.0, (**C**) 3.0, (**D**) 4.0 wt.% MMT. Solid lines: results of numerical analysis.

**Figure 8:**
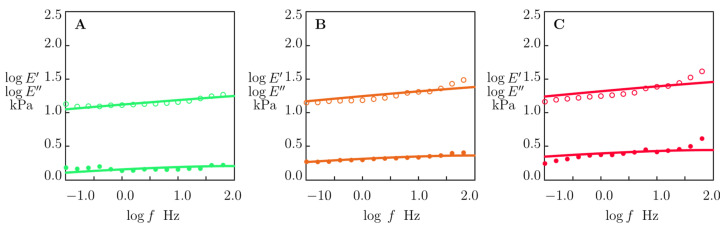
Storage modulus *E*′ (◦) and loss modulus *E*″ (•) versus frequency *f.* Symbols: experimental data in small-amplitude oscillatory tests at room temperature on alginate–MMT nanocomposite gels with various concentrations of MMT swollen in water with pH = 2. (**A**) 1.0, (**B**) 3.0, (**C**) 4.0 wt.% MMT. Solid lines: results of numerical analysis.

Figure 7 and Figure 8 demonstrate an acceptable agreement between the data and the results of the numerical simulation.

**Figure 9:**
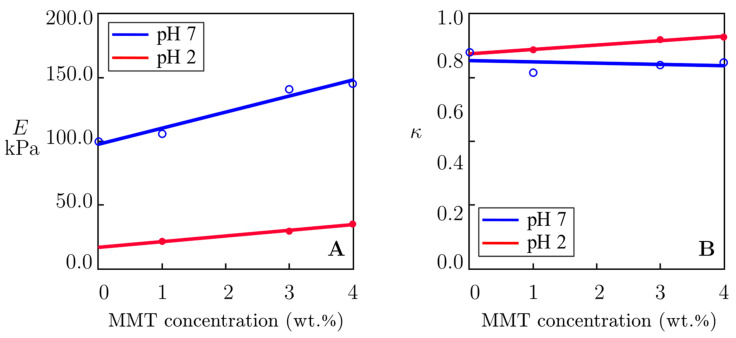
(**A**) Elastic modulus *E* and (**B**) coefficient *κ* versus MMT concentration. Symbols: treatment of experimental data at room temperature on alginate–MMT nanocomposite gels swollen in water with pH = 7 and pH = 2. Solid lines: results of numerical simulation.

The effect of mass fraction of MMT *c* on the parameters *E* and κ is illustrated in Figure 9, where the data are fitted by the linear functions

(5) $$E=E_{0}+E_{1}c,\;\;\kappa =\kappa_{0}+\kappa_{1}c$$

with the coefficients determined by the least squares method.

The slopes of the graphs depicted in Figure 9A equal 12.61 at pH = 7 and 4.46 at pH = 2, which means that the effect of MMT on the elastic modulus of nanocomposite gels is substantially (by about three times) stronger at pH = 7 than at pH = 2. Figure 9B shows that the coefficient κ (the ratio of the concentration of transient bonds to the total concentration of bonds between chains) is practically independent of the concentration of MMT, and its value is close to 0.8. Weak deviations of κ from the constant value can be explained by inconsistencies in measurements.

## 3. Discussion

The alginate–MMT nanocomposite hydrogels were prepared with various concentrations of MMT clay. By using the DMA technique, the interactions between MMT platelets and alginate chains in the gels swollen at pH = 7 and pH = 2 were investigated.

The frequency-sweep mode of small-amplitude oscillatory tests revealed a monotonical rise in both storage modulus *E*′ and loss modulus *E*″ with concentration of MMT at both pH values under investigation (Figure 4), indicating an increase in the elasticity and viscoelasticity of the nanocomposite gels.

The fact that the storage modulus *E*′ and the loss modulus *E*″ of the alginate–MMT nanocomposite gels exceed those of the neat alginate hydrogel (Figure 5) confirms the formation of cross-links between alginate chains and MMT platelets at pH = 2 and pH = 7 [28] (Figure 9A). A decrease in pH from pH = 7 to pH = 2 leads to a strong decrease in the storage and loss moduli (Figure 5), which can be explained by the breakage of weak bonds between chains at pH = 2 [41].

A model with two adjustable parameters was used to fit the experimental data obtained from small-amplitude oscillatory tests (Figure 7 and Figure 8). The effects of pH and concentration of MMT platelets on these quantities are illustrated in Figure 9.

Figure 9A shows that the elastic modulus *E* increases noticeably with concentration of MMT at pH = 7, but this growth becomes less pronounced at pH = 2. Presuming *E* to be proportional to the total number of bonds between chains, it can be concluded that the concentration of cross-links in the polymer network is reduced when the nanocomposite gel is swollen at pH = 2. In our previous study [14], it was shown that some egg-box bonds between alginate chains formed by Ca^2+^ ions were broken at low pH.

To explain why the effect of concentration of MMT on the elastic modulus becomes less pronounced at pH = 2, it is supposed that the structure of a polymer network in alginate–MMT nanocomposite gels changes dramatically when pH decreases from pH = 7 (which is strongly above the pK_a_ of alginate chains) to pH = 2 (which is below pK_a_).

At pH = 7, all the carboxyl groups attached to alginate chains are ionized, while all the MMT platelets in the gel have positive charges at their edges and negative charges at their surfaces [42]. Physical cross-links between the chains and the platelets are formed due to electrostatic interactions between negatively charged COO^−^ groups of chains and positively charged edges of the platelets.

At pH = 2, all the carboxyl groups become neutral. Physical bonds between the alginate chains and the clay platelets are formed due to hydrogen bonds between COOH groups attached to the chains and hydroxyl groups OH at the surfaces of clay platelets [43] (see Figure 6, where the interactions between MMT platelets and alginate chains are represented schematically).

The decrease in pH from pH = 7 to pH = 2 causes not only unzipping of some egg-box bonds between alginate chains but also phase transition in the bonds that link alginate chains with MMT platelets (Figure 6C). Both effects increase inhomogeneity in the nanocomposite gels, which is observed as an increase in the measure of inhomogeneity from Σ = 6.9 at pH = 7 to Σ = 13.5 at pH = 2. Replacement of “strong” electrostatic interactions between the chains and platelets at pH = 7 with “weak” hydrogen bonds results in a decay in the influence of concentration of MMT platelets on the elastic modulus *E* in accordance with the observations reported in Figure 9A.

According to Figure 9B, the coefficient κ (the ratio of the concentration of transient bonds to the total concentration of bonds between chains) is practically independent of the concentration of MMT. This result is rather unexpected, and several possible explanations for it can be provided. One of them is that the strength of bonds formed by egg-boxes between alginate chains is close to the strength of bonds formed between alginate chains and MMT platelets. In other words, when a gel is immersed into water with pH = 2, the number of unzipped egg-box bonds and the number of broken bonds between alginate chains and clay platelets decrease proportionally to each other (Figure 6C).

## 4. Materials and Methods

### 4.1. Materials

The sodium alginate from brown algae was purchased from Acros Organics (Geel, Belgium). Cloisite Na^+^, a natural and commercial montmorillonite (MMT) enriched with sodium, was acquired from Southern Clay Products (Gonzales, TX, USA). Calcium chloride (CaCl_2_) was provided by Merck (Burlington, MA, USA). Hydrochloric acid (HCl), 37% (*v*/*v*), was supplied by VWR International (Rosny-sous-Bois, France). Deionized water was used in the preparation of hydrogels and in all measurements.

### 4.2. Modification of MMT

MMT was modified in order to increase the gallery spacing between the platelets and to allow for a more effective mixing of the polymer with the MMT clay. First, 1 g of MMT powder was slowly added to 50 mL pre-heated deionized water at 80 °C under magnetic stirring at a speed of 500 rpm. After 4 h of stirring, the resulting MMT suspension was left to settle for 24 h. Then, the MMT suspension was ultrasonicated for 15 min, followed by centrifugation for 15 min at 4000 rpm. The resulting sediment was washed with deionized water and centrifuged again for 15 min at 4000 rpm. Washing operations were repeated until the supernatant became clear. The washed sediment was dried at 40 °C for 3 days, and then ground by hand into a fine powder for further processing [38,44].

### 4.3. Preparation of Hydrogels

A series of ionically cross-linked alginate–MMT hydrogels were prepared with various concentrations of MMT and a constant amount of CaCl_2_ by a method described previously in Ref. [14]. First, the pH of a 1 wt.% alginate solution (molecular weight of 216.12 kDa, G:M ratio 30:70) was reduced to 3.5 using HCl. This results in a decrease in the ionization degree of carboxyl groups along the alginate backbone. Then, 1, 3, and 4 wt.% of modified MMT powder was slowly added to the alginate solution and stirred until a homogeneous mixture was obtained. The alginate–MMT mixture was poured into a mold, and then a CaCl_2_ (0.5 M) solution was added. The mixture was left for 3 days to finalize the cross-linking procedure. Thereafter, the remaining unreacted moieties were removed by immersing hydrogels overnight in water with pH = 7.

DMA tests were conducted on swollen hydrogels in aqueous pH = 7 or pH = 2 (see Section 4.6). For XRD test, the gel with 2 wt.% MMT and, for FTIR test, the gel with 1, 3, and 4 wt.% MMT was dried at a temperature *T* = 30 °C for two days.

### 4.4. ATR-FTIR

ATR-FTIR measurements were conducted to assess the interactions between MMT platelets and alginate chains using a Spectrum One spectrometer from Perkin Elmer (Waltham, MA, USA). The analysis was performed in transmittance mode, employing a zinc selenide crystal with a resolution of 16 cm^−1^ and conducting 128 scans per measurement across the 500 to 4000 cm^−1^ range.

### 4.5. X-ray Diffraction

X-ray diffraction using an Empyrean diffractometer from PANalytical (Almelo, The Netherlands) with Cu Kα radiation at 45 kV and 40 mA was employed to explore the effect of ionic gelation with CaCl_2_ on the extent of MMT intercalation/exfoliation. The measurement range was between 2 and 17°.

### 4.6. Mechanical Tests

Small-amplitude compression oscillatory tests were performed to measure the storage modulus *E*′ and the loss modulus *E*″ by means of DMA Q800 V20.9 (TA Instruments, New Castle, DE, USA). The strain amplitude was 0.5% and frequency *f* was between 0.1 and 60 Hz at room temperature (*T* = 22 °C).

The DMA Q800 V20.9 (TA Instruments, New Castle, DE, USA) was employed to evaluate the storage modulus (*E*′) and loss modulus (*E*″) through small-amplitude compression oscillatory tests. These tests involved a strain amplitude of 0.5% and a frequency (*f*) ranging from 0.1 to 60 Hz, conducted at room temperature (22 °C).

First, the gels immersed in an aqueous solution with pH = 7 were assessed by measuring their storage modulus (*E*′) and loss modulus (*E*″). Then, the samples were immersed into an aqueous solution with pH = 2 for 2 days (to reach equilibrium, Figure S1) [14], and the DMA tests were repeated.

### 4.7. Statistical Analysis

In the small-amplitude compression oscillatory tests, three repetitions were conducted on disk-shaped samples with a diameter of 8.5 mm and a thickness of 4.5 mm, and their mean values were reported. The standard deviations of the data were less than 5% of their mean values.

## 5. Conclusions

A series of alginate–MMT nanocomposite hydrogels have been prepared with various concentrations of MMT platelets and CaCl_2_ as cross-linkers. Homogeneous nanocomposite gels are manufactured with MMT and CaCl_2_ up to 4 wt.% and 0.5 M, respectively. XRD analysis reveals an increase in the MMT gallery spacing from 12.03 to 14.92 Å. FTIR analysis demonstrates interactions of MMT platelets with alginate chains. The mechanical properties of nanocomposite gels were studied in aqueous solutions with pH = 7 and pH = 2 by means of DMA. It is shown that the storage modulus *E*′ and the loss modulus *E*″ increase with concentration of MMT platelets and decrease with pH. To explain these findings, the experimental data have been fitted by a model with only two adjustable parameters. It is demonstrated that the elastic modulus *E* grows linearly with MMT concentration at both pH values under investigation, but the increase in *E* is weaker at pH = 2. This finding is explained by replacement of electrostatic interactions at pH = 7 with hydrogen bonds at pH = 2 between MMT platelets and alginate chains. This phase transition is accompanied by a pronounced growth in the inhomogeneity of the polymer network (characterized by the measure of inhomogeneity Σ). It is shown that alginate–MMT nanocomposite gels preserve their integrity while being swollen in water with pH = 2, which implies that these hydrogels can be used as a drug carrier in environments with low pH.

## Figures and Tables

**Table 1:** FTIR peaks and corresponding functional groups in MMT, alginate, and alginate–MMT nanocomposite.

	Asymmetric Stretching Vibrations of Carboxylate Groups	Symmetric Stretching Vibrations of Carboxylate Groups	Oxygen Stretching	Stretching Vibration of Hydroxyl Groups	Bending Vibration of H–O–H	Stretching Vibration of Si–O–Si Bonds
MMT	-	-	-	3624 cm^−1^	1637 cm^−1^	1025 cm^−1^
Alginate	1595 cm^−1^	1405 cm^−1^	1019 cm^−1^	3268 cm^−1^	-	-
1 wt.% MMT	1605 cm^−1^	1422 cm^−1^	1030 cm^−1^	3318 cm^−1^	-	-
3 wt.% MMT	1610 cm^−1^	1429 cm^−1^	1017 cm^−1^	3363 cm^−1^		-
4 wt.% MMT	1611 cm^−1^	1436 cm^−1^	1031 cm^−1^	3381 cm^−1^	-	-

## Data Availability

The data that support the findings of this study are available from the corresponding author, H.M., upon reasonable request.

## Supplementary Materials

The following supporting information can be downloaded at: https://www.mdpi.com/article/10.3390/molecules29010244/s1, Figure S1: Degree of swelling *Q* versus time *t* for alginate hydrogels in aqueous solutions with pH = 7.
