# Supplementary material for: The Effect of pH on the Viscoelastic Response of Alginate–Montmorillonite Nanocomposite Hydrogels
Source: Molecules. 2024 Jan 2;29(1):244. doi: 10.3390/molecules29010244 (PMC10780325; doi:10.3390/molecules29010244)
Supplement: Supplementary file 1 [file molecules-29-00244-s001.zip › molecules-2725552-supplementary.pdf]

Supplementary material

The Effect of pH on the Viscoelastic Response of Alginate-Montmorillonite Nanocomposite Hydrogels

Haniyeh Malektaj [1,*], Aleksey D Drozdov [1], Elham Fini [2] and Jesper de Claville Christiansen [1]

[1] Department of Materials and Production, Aalborg University, Fibigerstraede 16, 9220 Aalborg, Denmark

[2] School of Sustainable Engineering and Built Environment, Arizona State University, 660 S College Ave, Tempe, AZ 85281, USA

* Correspondence: haniyehm@mp.aau.dk

To assess the characteristic time for equilibration of samples before mechanical tests, swelling tests were performed on as-prepared hydrogels immersed into aqueous solution with pH = 7. Experimental data are depicted in Figure S1, where the degree of swelling *Q* is plotted versus the immersion time *t*. This figure shows that 2 days of immersion into water is sufficient to reach the equilibrium.

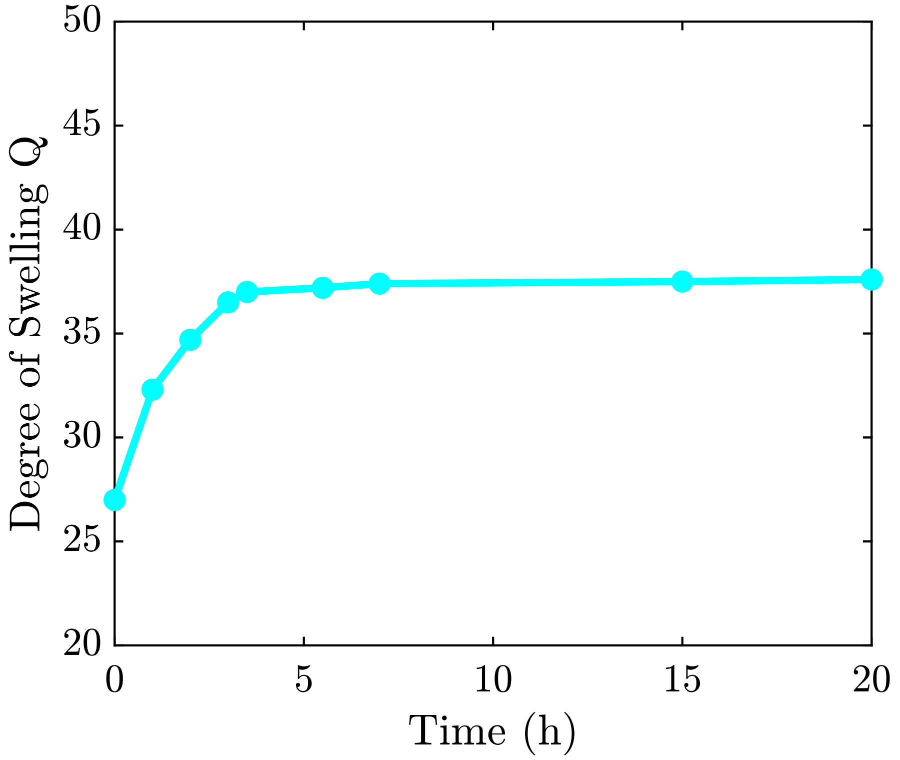

Figure S1. Degree of swelling *Q* versus time *t* for alginate hydrogels in aqueous solutions with pH = 7.
